# Multi-target immunofluorescence by separation of antibody cross-labelling via spectral-FLIM-FRET

**DOI:** 10.1038/s41598-020-60877-8

**Published:** 2020-03-02

**Authors:** Sumeet Rohilla, Benedikt Krämer, Felix Koberling, Ingo Gregor, Andreas C. Hocke

**Affiliations:** 1 PicoQuant Innovations GmbH, Rudower Chaussee 29 (IGZ), 12489 Berlin, Germany; 2 PicoQuant GmbH, Rudower Chaussee 29 (IGZ), 12489 Berlin, Germany; 3 Third Institute of Physics, Georg-August-University, Friedrich-Hund-Platz 1, 37077 Göttingen, Germany; 4 Charité – Universitätsmedizin Berlin, corporate member of Freie Universität Berlin, Humboldt-Universität zu Berlin, and Berlin Institute of Health, Department of Internal Medicine/Infectious Diseases and Respiratory Medicine, Charitéplatz 1, 10117 Berlin, Germany

**Keywords:** Fluorescence imaging, Immunological techniques

## Abstract

In biomedical research, indirect immunofluorescence labelling by use of primary and secondary antibodies is central for revealing the spatial distribution of multiple cellular antigens. However, labelling is regularly restricted to few antigens since species variation of primary and corresponding secondary antibodies is limited bearing the risk of unspecific cross-labelling. Here, we introduce a novel microscopic procedure for leveraging undesirable cross-labelling effects among secondary antibodies thereby increasing the number of fluorophore channels. Under cross-labelling conditions, commonly used fluorophores change chemical-physical properties by ‘Förster resonance energy transfer’ leading to defined changes in spectral emission and lifetime decay. By use of spectral fluorescence lifetime imaging and pattern-matching, we demonstrate precise separation of cross-labelled cellular antigens where conventional imaging completely fails. Consequently, this undesired effect serves for an innovative imaging procedure to separate critical antigens where antibody species variation is limited and allows for multi-target labelling by attribution of new fluorophore cross-labelling channels.

## Introduction

**Figure 1:**
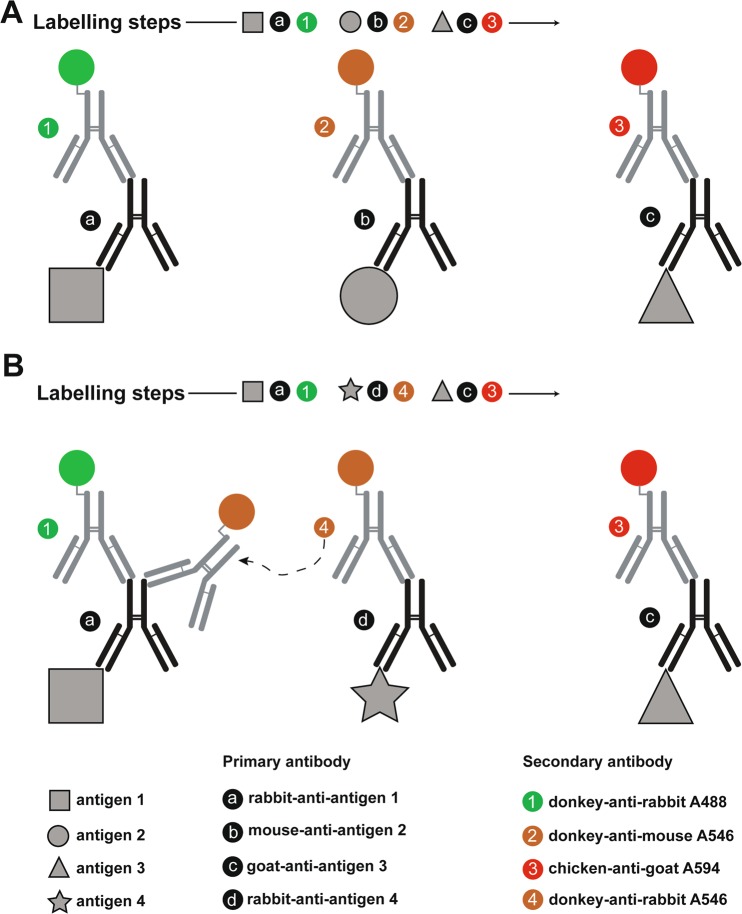
Non-cross- and cross-labelling in indirect immunofluorescence by secondary antibodies. Shown is a typical sequential labelling procedure used for indirect immunofluorescence (IMF) of three target antigens to demonstrate the resulting labelling in two scenarios. (**A**) Regular state: three different primary antibodies (ABs) origin from rabbit, mouse and goat bind to antigens 1, 2 and 3, respectively. For IMF detection, fluorophore-tagged secondary ABs (“donkey-anti-rabbit Alexa488”, “donkey-anti-mouse Alexa546” and “chicken-anti-goat Alexa594”), which are in origin different from that of targeted primary AB, will not show any cross-labelling. (**B**) Undesirable cross-labelling: primary ABs for antigens 1, 3 and 4 are originated from rabbit and goat. In this scenario, conjugation of secondary AB for IMF will lead to cross-labelling of rabbit primary AB for antigen 1 with “donkey-anti-rabbit Alexa546” leading to false-positive attribution of “donkey-anti-rabbit Alexa546” AB on antigen 1, bearing the risk of misinterpretation of results such as (co-)localization, spatial distribution, or even molecule-molecule interaction. For easy illustration purposes, a stoichiometry of 1:1 between primary and secondary AB is shown here, however in general, there are on an average more than one fluorophore attached to secondary AB. Similarly, depending on the available binding sites, the number of secondary AB bound to primary AB could vary between 2 to 5.

The technique of indirect immunofluorescence (IMF) by labelling antigens with primary and secondary antibodies (ABs) is still one of the most extensively used methods in microscopy to reveal the spatial distribution of molecules of interest in cells or tissues, thereby excellently serving to reach a deeper understanding of biological processes^1^. Next to careful planning of fixation, labelling sequences and proper AB controls, a further crucial aspect of IMF is the need for a stringent selection of primary and secondary AB pairs to avoid false-positive immunolabelling due to species overlap, especially if multiple antigens are targeted^2,3^. At best, primary ABs should originate from different species and combined with corresponding secondary ABs, all raised in one disparate host animal species or, at least, differing from the species origin of all primary and other secondary ABs (Fig. 1). Although a broad species panel of primary ABs is principally available (e.g. mouse, human, rabbit, rat, goat, chicken, sheep, guinea pig, hamster, bovine, donkey, dog, camelid, cat, pig etc.), practically, most specific and well-performing primary ABs for important target bio-molecules originate from mouse, rabbit, rat, or goat. Thus, if typical multi-target approaches include the repetitive labelling of organelles or structural proteins with established primary ABs, the combination of further molecules of interest is increasingly restricted by AB species overlap. A series of studies demonstrated how to overcome such problems for double immunolabelling using ABs produced in same species^4–9^. However, all these will finally still not completely avoid false-positive cross-labelling of desired antigens, bearing the risk of misinterpretation regarding (co-)localization, spatial distribution, or even interaction of molecules^10–13^.

But, what if such false-positive cross-labelling can be used as an advantage by generating new, independent fluorescence analysis channels? Indeed, this would transform cross-labelling due to species overlap of secondary ABs into a useful tool to expand IMF towards reliable and unambiguous multi-target labelling. A prerequisite to achieve this advantage is that single- as well as cross-labelled structures exhibit significant differences in their fluorescence characteristics allowing for precise separation and channel attribution. Like almost any dual fluorophore labelled bio-molecule with considerable overlap between their emission and absorption spectra, interacting fluorophores on ABs might change their chemical-physical properties by the so called ‘Förster resonance energy transfer’ (FRET), which was already nicely shown by previous work of Holzapfel *et al*.^14^. FRET occurs when fluorophores on cross-labelled ABs reach a physical proximity <10 nm resulting in a shift of two important characteristics, the fluorescence lifetime decay as well as the spectral emission. Therefore, by a combinatorial approach of measuring fluorescence lifetime decay as well as spectral information, cross-labelled structures could be precisely identified and separated from single labelled molecules. In a similar approach, we already demonstrated in the study of Niehörster *et al*. that the combinatorial use of fluorescence lifetime decay as well as spectral information is suitable for separation of up to nine different fluorophores in a cellular environment^15^. Here, we adapt this method to demonstrate that the use of cross-labelling due to species overlap enables us to introduce new specific fluorescence analysis channels. To proof this hypothesis, we adapted a sequential IMF labelling protocol on A549 cells and selected appropriate fluorophore pairs on interacting secondary ABs to achieve FRET effects on cross-labelled cellular target molecules. Additionally, simultaneous acquisition and further processing of fluorescence lifetime decays as well as emission spectra necessitate proper hardware configuration and is again accomplished by means of time-domain spectrally resolved fluorescence lifetime imaging microscopy (sFLIM)^15^. As we and others already demonstrated, sFLIM combines the advantage of two non-filter based and independent confocal imaging modalities^15–17^. Multi-channel spectral imaging and time-resolved FLIM are combined for quantification of FRET interactions with high precision and single molecule sensitivity to detect and separate multiple distinct fluorescence channels^18–22^. Thus, we have built an 8-channel sFLIM detection system for quantification and verification of FRET signatures resulting from interaction of fluorophore labels on secondary ABs, which should demonstrate the capability to distinguish cross- and single-labelled target molecules in a typical biological IMF scenario. For data analysis we used an already established pattern-matching algorithm based on linear-unmixing which takes into account emission spectra as well as nanosecond fluorescence decays^15^.

In this proof-of-concept study, we could demonstrate how multiplexing in standard IMF gets possible with cross-labelled secondary ABs by the combination of multi-dimensional sFLIM and pattern-matching based unmixing achieving a minimum of bleed-through/cross-talk of fluorescence signals among different unmixed fluorescence analysis channels.

## Results

### Conventional channel mode imaging is insufficient to separate cross-labelled secondary antibodies

**Figure 2:**
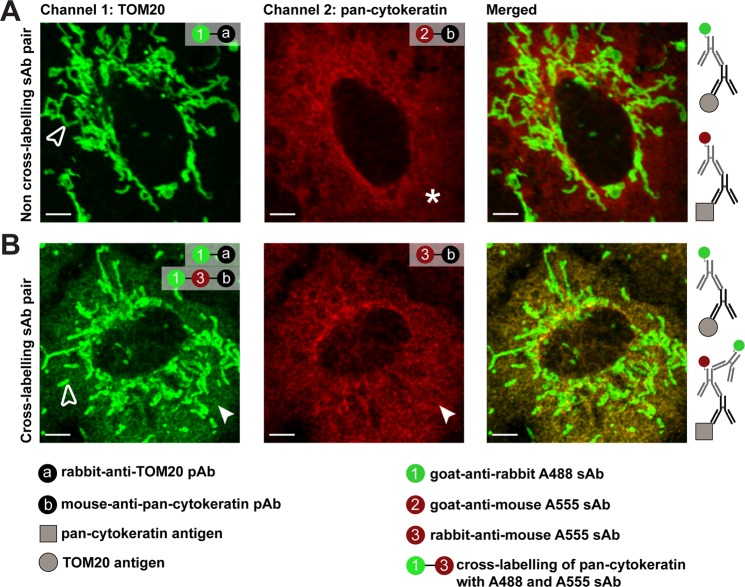
Channel mode imaging fails to separate cross-labelling in indirect immunofluorescence. (**A**) A549 cells were used to label two antigens, TOM20 and pan-cytokeratin, with a pair of non-cross-labelling secondary antibody (AB) (“goat-anti-rabbit Alexa488”, open arrowhead; “goat-anti-mouse Alexa555”, asterisk) resulting in correct-labelling and separation by channel mode imaging. (**B**) In contrast, labelling of TOM20 and pan-cytokeratin with a pair of cross-labelling AB species (“goat-anti-rabbit Alexa488”, open arrowhead; “rabbit-anti-mouse Alexa555”, closed arrowhead) resulted in false-positive attribution of pan-cytokeratin in TOM20 channel indicated by green cytosolic signal (closed arrowhead). Conventional channel mode imaging is demonstrated here as insufficient for eliminating channel cross-talk by cross-labelling of secondary ABs, even by using proper emission bandpass filters (520/14 nm and 593/20 nm). Representative images from three independent experiments are shown; scale bars 5 µm.

In standard IMF, primary and secondary ABs are used to label different cellular antigens. By use of non-cross-labelling secondary ABs, conventional channel mode imaging shows a clear separation of fluorescence analysis channels if emission bandpass filters are properly configured. We first demonstrate A549 cells labelled for mitochondrial TOM20 and the structural protein pan-cytokeratin without any cross-labelling between secondary ABs (Fig. 2A). For this setting, channel mode imaging, either in wide-field or confocal mode, is fully sufficient to separate and allocate the two cellular antigens precisely. However, in case of species overlap leading to cross-labelling of one secondary AB with another, which is demonstrated here by “goat-anti-rabbit Alexa488” labelling of TOM20 *and* the secondary AB “rabbit-anti-mouse Alexa555”, a false-positive signal for pan-cytokeratin appears in the TOM20 channel indicating cytosolic co-localization of both antigens (Fig. 2B and see Supplementary Fig. 1 for labelling protocol). If such choices of secondary ABs cannot be avoided, even the proper setting of emission band pass filters is insufficient for signal separation showing the obvious limitation of conventional filter-based microscopic methods.

### Cross-labelling between secondary antibodies leads to FRET

**Figure 3:**
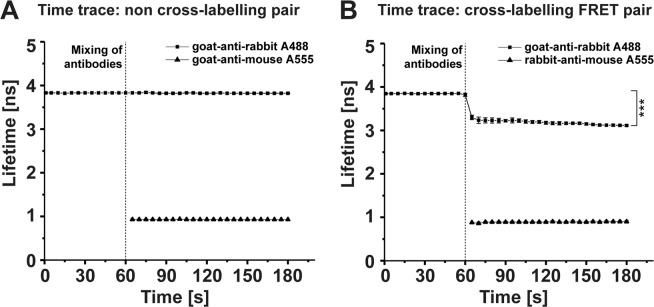
Cross-labelling of secondary antibodies leads to FRET. Two pairs of fluorophore-conjugated secondary antibodies (ABs) were mixed and measured for lifetime changes. (**A**) Non-cross-labelling ABs (“goat-anti-rabbit Alexa488” and “goat-anti-mouse Alexa555”) do not show any change of their average fluorescence lifetimes of about 3.83 ± 0.10 ns and 0.93 ± 0.10 ns, respectively. (**B**) Using cross-labelling secondary ABs (“goat-anti-rabbit Alexa488” and “rabbit-anti-mouse Alexa555”) leading to FRET, a significant shift of the donor fluorescence lifetime (Alexa488) from 3.84 ± 0.01 ns to 3.18 ± 0.04 ns (Δ*τ*_*int*_ = 0.66 ns, intensity weighted lifetime) was measured. Quantification of donor and acceptor fluorescence lifetime is given as mean ± SD from three independent experiments; ^***^p < 0.001.

Since conventional channel mode imaging is insufficient to separate fluorophore emission of cross-labelled secondary ABs, we reasoned that the alteration of the photophysical properties of the used fluorophores caused by FRET will be suitable to address this issue^14^. As FRET pairs, we again chose the ABs “goat-anti-rabbit Alexa488” (donor) and “rabbit-anti-mouse Alexa555” (acceptor) as well as “goat-anti-rabbit Alexa488” and “goat-anti-mouse Alexa555” as non-cross-labelling negative control. Respective fluorescence lifetimes were quantified by standard time-correlated single-photon counting based fluorescence lifetime imaging microscopy (TCSPC-FLIM) and using the SymPhoTime 64 (PicoQuant, Germany) analysis software. As a first step, we obtained fluorescence lifetimes of free secondary ABs in aqueous solution (Supplementary Table 1A). Afterwards, non- as well as cross-labelling AB pairs were mixed, the AB concentrations were measured by fluorescence correlation spectroscopy (FCS) and, fluorescence lifetimes were quantified by TCSPC-FLIM in aqueous solution (Fig. 3). As hypothesised, no change in donor fluorophore lifetime was revealed for the non-cross-labelling AB pair (Fig. 3A), but a significant decrease in the donor fluorophore lifetime was observed directly after AB mixing at time *t *= 60 s, clearly indicating FRET (Δ*τ*_*int*_ = 0.66 ns, difference between unquenched and quenched donor fluorophore intensity weighted lifetime) for the cross-labelling AB pair (Fig. 3B). This analysis demonstrates that fluorophores conjugated to cross-labelling secondary ABs undergo FRET interaction, which might be suitable for separation in a cellular IMF scenario.

### Separation and new channel attribution for cross-labelled antibodies by sFLIM-FRET

**Figure 4:**
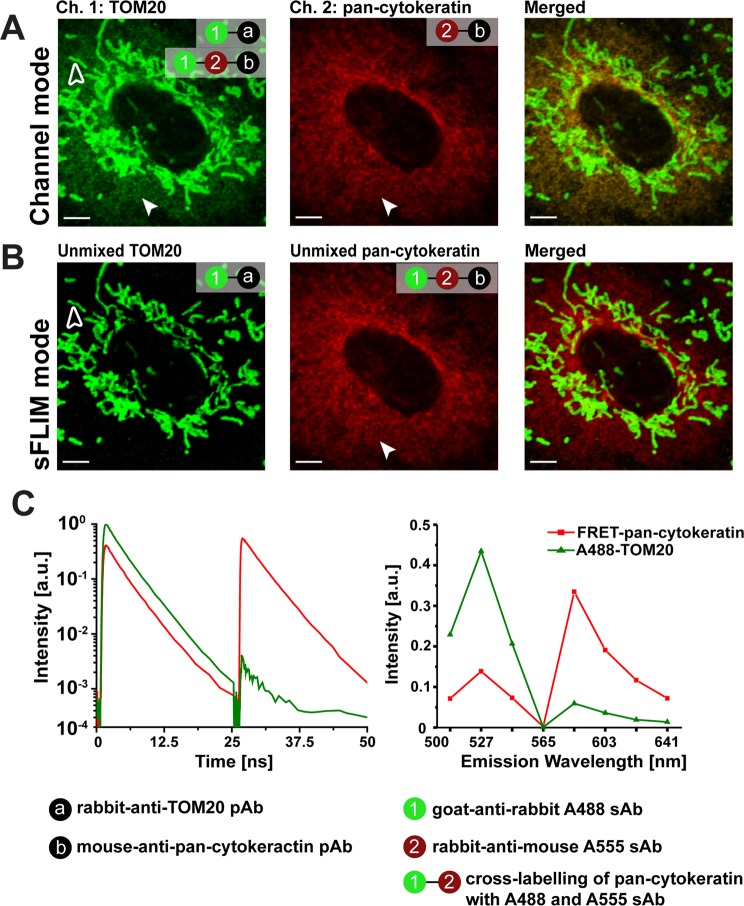
Spectral-FLIM-FRET for separation of cross-labelling in dual antigen indirect immunofluorescence. A549 cells were immunolabelled for TOM20 (with “goat-anti-rabbit Alexa488”) and pan-cytokeratin (with “rabbit-anti-mouse Alexa555”) antigen leading to single labelling of TOM20 and cross-labelling of pan-cytokeratin. (**A**) Conventional channel mode imaging is insufficient for eliminating the attribution of pan-cytokeratin (arrowhead) to the TOM20 channel (open arrowhead). (**B**) The same cells were measured by spectral-FLIM. Pattern based unmixing provides pixelwise the fluorescence contributions for Alexa488 labelled TOM20 (open arrowhead) and cross-labelled pan-cytokeratin (closed arrowhead). Each labelled antigen structure now is clearly attributed to separate analysis channel. (**C**) Fluorescence decays (left panel) corresponding to two excitation laser pulses (485 nm and 561 nm) and emission spectra (right panel) were used as combined reference patterns for unmixing Alexa488 labelled TOM20 and FRET antibody pair labelled pan-cytokeratin (Alexa488 and Alexa555). Representative images from three independent experiments are shown; scale bars 5 µm.

As demonstrated, cross-labelling secondary ABs undergo FRET interaction, which we now aimed to use as advantage in IMF for creating new fluorescence analysis channels by sFLIM. To demonstrate the potential of sFLIM for resolving undesired cross-labelling, we again performed dual antigen IMF as introduced in Fig. 2B. As a first step, we performed multi-dimensional fluorescence imaging of dual antigen IMF sample by sFLIM system (Supplementary Fig. 2, see Materials and Methods for system details). Using this system, we verified whether cross-labelling of pan-cytokeratin with secondary AB pairs resulted in FRET effects or not. A donor-only sample where pan-cytokeratin was cross-labelled with “goat-anti-rabbit Alexa488” and “unconjugated rabbit-anti-mouse” AB was used as control for FRET quantification on pan-cytokeratin. Fluorescence decays obtained by sFLIM measurements of FRET AB pair and donor-only labelled control samples were fitted to obtain quenched and unquenched donor lifetime using the SymPhoTime 64 software (Supplementary Table 1B). As hypothesized, we revealed that close binding of cross-labelling AB pairs on pan-cytokeratin indeed resulted in FRET (Δ*τ*_*int*_ = 0.47 ns, difference between unquenched and quenched donor fluorophore intensity weighted lifetime). Next, as a prerequisite for sFLIM based quantitative separation, we first demonstrate again that separation of fluorescence signals from cross-labelled pan-cytokeratin is impossible by conventional filter-based microscopy (Fig. 4A). However, by using appropriate reference patterns corresponding to each labelled antigen and a pattern-matching algorithm for quantitative estimation of fluorescence contribution of both antigens per pixel, we could precisely attribute single-labelled TOM20 and cross-labelled pan-cytokeratin into separate analysis channels (Supplementary Fig. 3 and Fig. 4B). A reference pattern is defined as a characteristic fluorescence signature of antigen labelling combining both information, emission spectra as well as time-resolved fluorescence decays. The dip in the recorded reference emission spectra is due to the use of the notch filter used to avoid detection of scattered light from 561 nm excitation wavelength. For optimal unmixing of fluorescence signals of both labelled antigens, we used reference patterns obtained from “goat-anti-rabbit Alexa488” labelled TOM20 sample, whereas pan-cytokeratin FRET reference pattern was obtained from dual antigen IMF sample (Fig. 4C, see Material and Methods for details). A direct comparison of the resulting merged multi-colour fluorescence images obtained by channel mode (Fig. 4A, merged) and sFLIM (Fig. 4B, merged) as well as the comparison of line profiles of selected region (Supplementary Fig. 5A) clearly demonstrates the advantage gained by multi-dimensional sFLIM acquisition followed by linear-unmixing based pattern-matching data analysis.

### sFLIM-FRET enables triple antigen IMF by using just two fluorophores

**Figure 5:**
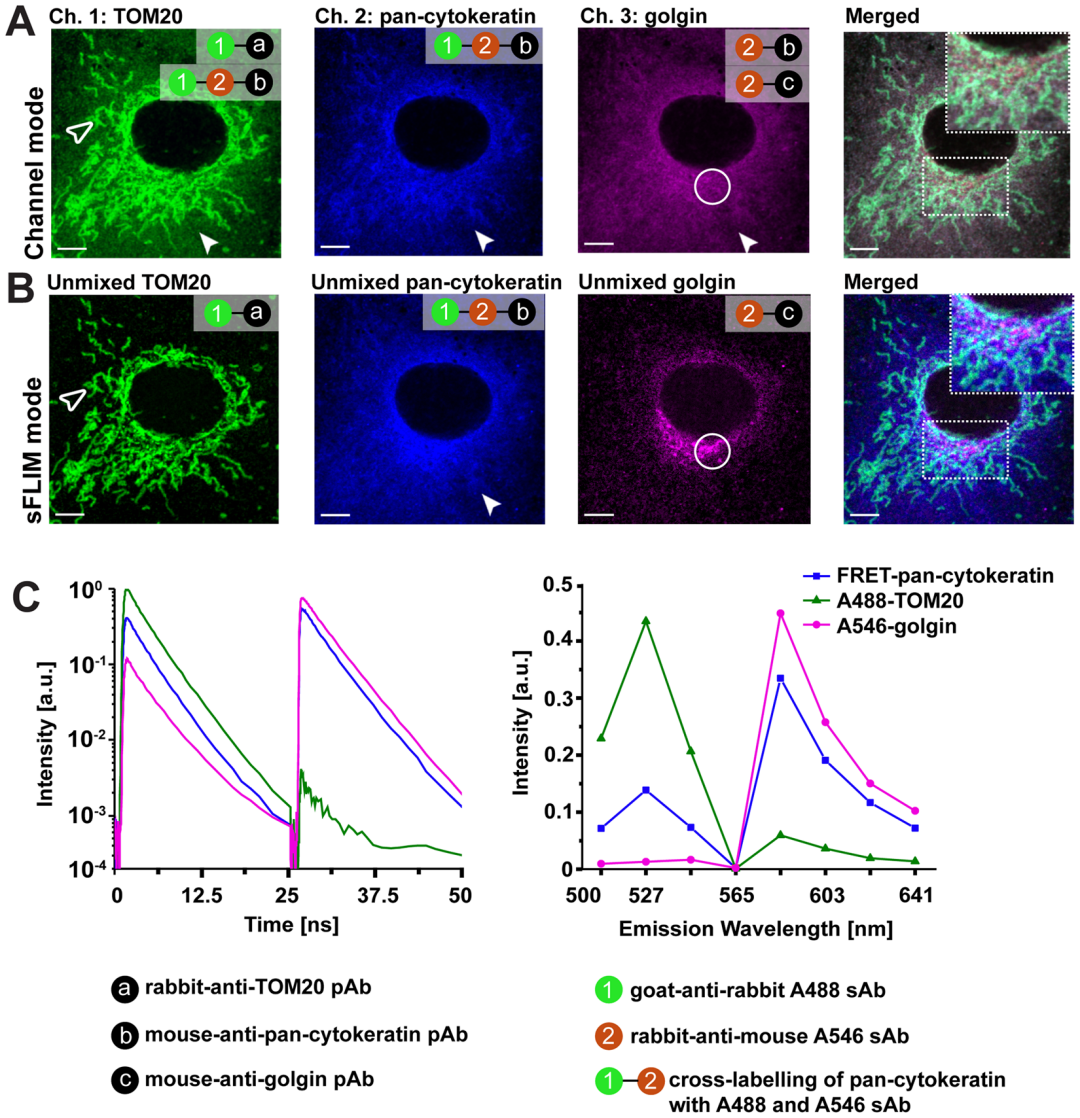
Spectral-FLIM-FRET for separation of cross-labelling in triple antigen indirect immunofluorescence. A549 cells were immunolabelled for three different cellular antigens with just two primary (mouse, rabbit) and secondary (“goat-anti-rabbit Alexa488”; “rabbit-anti-mouse Alexa546”) antibody (AB) species types. Following a sequential labelling scheme, single labelling of TOM20 (“goat-anti-rabbit Alexa488”), golgin (“rabbit-anti-mouse Alexa546”) and cross-labelling of pan-cytokeratin (“goat-anti-rabbit Alexa488”; “rabbit-anti-mouse Alexa546”) was achieved. (**A**) Conventional channel mode imaging was insufficient for eliminating false-positive pan-cytokeratin (closed arrowhead) in the TOM20 channel and golgin channel. (**B**) The spectral-FLIM data acquisition and pattern-matching analysis allows to separate the fluorescence contributions of all three antigens into independent analysis channels: Different structures (TOM20 (open arrowhead), pan-cytokeratin (closed arrowhead), golgin (open circle)) are clearly visualized with a notable false-positive suppression (compare merged images of A and B). (**C**) Reference patterns, combining fluorescence decay (left panel, 485 nm and 561 nm excitation laser wavelength were used) and emission spectra (right panel) information, of Alexa488 labelled TOM20, Alexa546 labelled golgin as well as FRET AB pair labelled pan-cytokeratin used by pattern-matching algorithm for unmixing of their fluorescence contribution per pixel. Representative images from three independent experiments are shown; scale bars 5 µm.

To further demonstrate the potential of sFLIM for separation of cross-labelling, triple antigen IMF for TOM20, pan-cytokeratin and golgin was performed with a combination of two species for primary AB origin (mouse, rabbit) as well as two fluorophore-tagged secondary AB (“rabbit-anti-mouse Alexa546” and “goat-anti-rabbit Alexa488”). Following the proposed labelling sequence, pan-cytokeratin was positive for “rabbit-anti-mouse Alexa546”, TOM20 for “goat-anti-rabbit Alexa488”, and golgin for “rabbit-anti-mouse Alexa546” (Supplementary Fig. 4A). This procedure inevitably led to additional cross-labelling of pan-cytokeratin with “goat-anti-rabbit Alexa488” and subsequent FRET effects between interacting fluorophore-tagged AB. This was verified by extracting the mean lifetime from the fluorescence decays obtained from sFLIM measurements of cross-labelled (FRET control) and donor only (no–FRET control) labelled pan-cytokeratin sample (Supplementary Table 1C). Data indicates a clear pan-cytokeratin related FRET effect caused by fluorophore-tagged AB cross-labelling (Δ*τ*_*int*_ = 0.67 ns, difference between unquenched and quenched donor fluorophore intensity weighted lifetime). It is important to follow the correct labelling steps to achieve triple antigen IMF using two fluorophores, and we illustrate this in another exemplary scenario (Supplementary Fig. 4B). Next, we again demonstrate that filter-based channel mode imaging is insufficient for separating cross-labelled fluorescence signals from three labelled antigens (Fig. 5A). It can be seen that pan-cytokeratin is false-positively attributed in TOM20 and golgin channel, thereby completely covering the golgin signal. Taking advantage of multi-dimensional sFLIM followed by pattern-matching analysis, however, pixelwise quantitative separation of fluorescence contribution from all three immunolabelled antigens into different fluorescence analysis channels was possible, albeit the complexity due to spatial overlap of all antigens (Fig. 5B). The clear separation of fluorescence signal into different color channels is demonstrated very nicely in line profiles plots of selected regions in merged image (Supplementary Fig. 5B). For optimal unmixing results, reference patterns generated from single labelled TOM20 and golgin control samples were used, whereas it was appropriate to generate pan-cytokeratin reference pattern from triple antigen IMF samples (Fig. 5C). Bleed-through estimated from unmixed pan-cytokeratin channel to unmixed TOM20 and golgin channel were less than 2%. And, bleed-through of unmixed TOM20 and golgin channels into other unmixed channels were less than 1% (data not shown).

## Discussion

In this study, we present a novel imaging strategy suitable to leverage false-positive cross-labeling in IMF for precise multiple antigen detection. We describe a definitive labeling procedure and correct selection of controls as well as fluorophores serving as proper FRET pairs to perform sFLIM analysis for attribution of new fluorescence analysis channels corresponding to cross-labelled antigens. To demonstrate the power of this method, double and even triple antigen IMF was carried out by use of just two fluorophores, which would be impossible to separate by conventional channel mode imaging in cross-labelling scenarios. This advantage and precision become possible since the pattern-matching analysis of sFLIM data provides minimal bleed-through between unmixed fluorescence channels, as already demonstrated in our previous study where up to nine fluorophores were intra-cellularly separated^15^. Based on these findings we demonstrate herein, next to mere fluorophore separation, sFLIM allows for labelling of different cellular antigens with ABs raised in the same species.

The major advantage of this microscopic method and analysis is that IMF protocols with often well-established primary ABs can be maintained and require just adaption for the new labelling sequence of the secondary AB to get proper controls as well as efficient FRET effects on double labelled antigens. Consequently, no establishment of new IMF kits for avoiding false-positive cross-labelling is necessary, which saves time, costs and, most importantly, helps to avoid misinterpretation such as wrongly attributed co-localization or spatial distribution of cellular structures and proteins. Indeed, previous studies have already shown that it is possible to achieve double or multiple staining of target biomolecules with ABs derived from the same species. For instance, Tsurui *et al*. established a method for seven-colour analysis of immunofluorescence stained tissue with a labelling scheme consisting of monoclonal AB treated with avidin-biotin complex (ABC) in combination with seven distinct fluorophores^6^. Although, use of high ABC ratios allows for increased sensitivity, but at the same time bares the risk of increased non-specific background since avidin can bind to endogenous biotin in cells and tissues^3^. Additionally, the filter-based imaging setups used in these studies allow just for sequential excitation per dye group (blue, green, red) and data analysis requires additional dummy exposure images to remove false-positive contribution/bleed-through from shorter wavelength dye groups into red ones. In comparison, sFLIM can be carried out with simultaneous excitation due to pulse interleaved excitation (PIE) and multi-color acquisition and, even more important, provides better signal to noise ratios since both, decay and spectral information, are combined in the unmixing algorithm leading to images with minimal bleed-through and precise fluorophore channel attribution.

In another approach, researchers demonstrate the effective use of primary ABs raised in same species to visualize two antigens simultaneously^4,7,8^. However, these methods require an intermediate blocking step with AB F(ab) fragments for the first staining which results in higher background due to non-specific binding of blocking AB fragments, and consequently, decreased intensity of first labelling possibly due to over-blocking of AB on the first antigen^4^. Following a similar approach, Franzusoff *et al*. were able to demonstrate labelling of two antigens with two rabbit primary antibodies treated with “goat-anti-rabbit” and “mouse-anti-rabbit” F(ab) fragments and visualized using “rabbit-anti-goat FITC” and “rabbit-anti-mouse TRITC”, respectively^9^. However, a limitation of this method is that primary antibodies must be pre-treated in solution to avoid any cross-labelling between anti-rabbit AB fragments, which probably reduces the affinity of the primary ABs for their target antigens in addition to increased sample preparation time steps (three step labelling procedure instead of typical two step IMF). In total, the labelling scheme which we propose in this study, provides a novel solution to circumvent the problem of limited variability of primary AB species for IMF studies.

Although using FLIM for FRET analysis is technically demanding, this method is still recognized as the gold standard for quantitative FRET as demonstrated in the study by Pelet *et al*.^22^. Similar methods such as acceptor photo-bleaching or sensitized emission would be completely insufficient to identify cross-labelled antigens precisely. In the first approach, acceptor fluorophores of the entire cell would have to be bleached to identify areas of donor fluorophores (which are hidden among none-donor fluorophores), which would at the same time destroy the fluorescence of all acceptors (including non-cross-labelled). In the second approach, donor excitation for measuring sensitized emission would be probably useful to identify signals just in FRET positive areas, however, in a non-quantitative and spatially unprecise manner. This has a certain reason: normally, sensitized emission is used in scenarios where just FRET or non-FRET should be identified to infer a yes or no effect. However, the precise spatial separation of FRET and non-FRET fluorophores, where ‘correct’ and ‘false-positive’ cross-labelling has to be accurately distinguished, is not at all the domain of this approach. A more advanced method would be if the sensitized emission method is used in spectral mode where the donor/acceptor ratio can be measured at once. However, even spectral FRET alone is insufficient to quantitatively distinguish donor/acceptor fluorophores from non-donors/non-acceptors leading to a false attribution of fluorescence channels. Therefore, we used the time domain FLIM technique in this proof of concept study to quantify FRET effects due to cross-labelling ABs, in solution as well as in cellular IMF. FLIM was further combined with the spectral domain since the  former is necessary for separating fluorophores in more complex multiplex scenarios where fluorophore emission spectra are strongly overlapping. The benefit for the unmixing results has been demonstrated in our previous study, where it was shown that separation quality decreased substantially when spectral or lifetime information for unmixing were used separately^15^. In total, the inherent ability of the sFLIM method to resolve fluorophores with spectroscopically similar fluorescent signatures or even FRET of cross-labelling ABs, as shown here, potentially enhances the possible number of combinations of fluorophores which could be multiplexed, imaged and visualized simultaneously. This is supported by comparable studies which likewise demonstrate that sFLIM successfully enables FRET quantitation with high accuracy^23–25^.

While our study provides an easy to use labelling procedure leveraging FRET effects between cross-labelling ABs for generating additional fluorescence analysis channels, it is nevertheless important to consider that the labelling sequence and AB concentrations might affect the ratio of donor to acceptor molecules on the FRET pair labelled antigen. For instance, it is expected that FRET effects will be stronger for conditions where multiple acceptor molecules are available per donor molecule. We noticed similar increased FRET effects (or larger decrease in donor fluorophore lifetime) on pan-cytokeratin which could possibly be explained by the surplus of acceptor molecules in triple antigen IMF (see Supplementary Fig. 4A). Similar effects were also reported in two independent studies, where it was shown that presence of two acceptor molecules per single donor molecule leads to significant increase in FRET effects^26,27^. We also noticed that stronger FRET effects lead to better separation quality of cross-labelled antigen from single labelled antigen due to significant differences in their fluorescence signatures (data not shown). Furthermore, for schematic illustration we simplified the labeling stoichiometry to 1:1 between fluorophores and Abs as well as for secondary AB to single primary AB binding. However, several fluorophore molecules and secondary ABs with an unknown exact ratio must be considered to be attached respective binding partners (see Fig. 1, Supplementary Figs. 1 and 4). This fact leads to higher stoichiometry between fluorophores and ABs and is considered to potentially stabilize FRET effects as the probability of donor or acceptor fluorophore missing on the ABs is reduced. For a deeper analysis, measurements of molecular brightness e.g. by using FCS could be performed to determine the degree of labelling by comparing brightness of single and multi- fluorophore-tagged secondary ABs. In general, the success of the labelling approach described in this study greatly depends on whether the cross-labelling fluorophore ABs are FRET compatible or not. As shown by Holzapfel *et al*., use of Fab fragments, as opposed to full IgG molecules, will exhibit observable FRET on cross-labelled antigen, since the large size of full IgG molecules compared to Fab fragments leads to decreased interaction between donor and acceptor fluorophores^14^. This has to be taken into consideration. Another crucial aspect to be taken into account is the significant change in fluorescence lifetime values of fluorophore-tagged secondary ABs going from unfixed solution to fixed samples in cells of IMF (see Supplementary Table 1). This phenomenon is well known and shows again the dependency of fluorophore lifetime on the actual environment influenced by fixation, pH, mounting media, etc. In a study by Joosen *et al*., similar findings and necessary control experiments were suggested to account for such causes and effects^28^. Hence, it is an important pre-requisite to perform control studies and experiments to ensure FRET interaction among interacting AB as free dyes in solution, which is indeed easy to perform. While one might be tempted to use higher AB concentration to achieve stronger FRET effects, one must find, through trial and error experiments, optimal primary and secondary AB concentrations in addition to labelling specificity and efficiency to avoid artefacts due to any non-specific background staining. The choice of labelling sequence could also affect the number of acceptor molecules per donor molecule or even no FRET labelling on target antigen at all. For example, in triple antigen IMF, performing the TOM20 labelling first will result in cross-labelling of pan-cytokeratin with “rabbit-anti-mouse Alexa546” AB which makes it difficult to separate from golgin due to their similar spectral and fluorescence decay properties.

In essence, this new microscopic approach presented herein allows researchers to use the adversity of cross-labelling artefacts in IMF to their advantage and, at the same time, provides a general solution for multiplex IMF using AB originating in the same host animal species. Thus, researchers can develop individual labelling schemes based on already well-established primary ABs combined by using various commercially available fluorophores of secondary ABs which are proper FRET pairs (e.g. Cy3/Cy5 or ATTO488/ATTO565 etc.). Taken together, an adapted IMF labelling protocol in combination with sFLIM and pattern-matching analysis serves as innovative and proper method benefitting the entire research field where AB based IMF is carried out.

## Methods

### Antibodies

The primary AB against pan-cytokeratin and TOM20 were obtained from Santa Cruz Biotech (Germany). The anti-golgin primary AB and the Alexa Fluor^®^ conjugated secondary AB were purchased from ThermoFisher Scientific (Germany). The “unconjugated rabbit-anti-mouse” secondary AB was from Dianova (Germany).

### Cell culture and indirect immunofluorescence labelling

Human lung alveolar epithelial cell line A549 (ATCC, CCL-185) was cultured in Ham’s F12 medium (Biochrome, Berlin, Germany) supplemented with 10% fetal calf serum at 37 °C and 5% CO_2_. Cells were seeded on optical coverslips. After three washes with phosphate buffered saline (PBS), cells were fixed with 3% paraformaldehyde for 15 min at room temperature followed by three times washing with PBS. Afterwards, cells were permeabilized with 1.0% Triton X100 for 15 min. After washing with PBS, cells were blocked with AB diluent (20 ml PBS 0.01 M with 0.2 g BSA and 0.01 g Tween-20) and primary AB (2 μg/ml) were incubated overnight at 4 °C. After three times washing with PBS, cells were subjected to an overnight incubation with respective secondary AB at 4 °C. Immunolabelled cells were embedded in MOWIOL for 20 min at 4 °C.

### Labelling procedures for sFLIM-FRET analysis

Dual antigen IMF of cells for pan-cytokeratin and TOM20 with non-cross-labelling AB pair was performed with “goat-anti-mouse Alexa555” and “goat-anti-rabbit Alexa488” AB, respectively. Dual labelling of pan-cytokeratin and TOM20 with cross-labelling AB pair was performed with “rabbit-anti-mouse Alexa555” and “goat-anti-rabbit Alexa488” AB (Supplementary Fig. 1). Pan-cytokeratin cross-labelled with “unconjugated rabbit-anti-mouse” and “goat-anti-rabbit Alexa488” AB served as a zero FRET (donor-only) control sample. Additionally, single antigen IMF samples were prepared to obtain reference patterns from TOM20 labelled with “goat-anti-rabbit Alexa 488” as well as pan-cytokeratin cross-labelled with FRET AB pair (“rabbit-anti-mouse Alexa555” and “goat-anti-rabbit Alexa488”).

For triple antigen IMF, cells were sequentially labelled to achieve single labelling of TOM20 with “goat-anti-rabbit Alexa488” AB and golgin with “rabbit-anti-mouse Alexa546” AB as well as cross-labelling of pan-cytokeratin with “goat-anti-rabbit Alexa488” and “rabbit-anti-mouse Alexa546” FRET AB pair (Supplementary Fig. 4A). As described above, a zero FRET (donor-only on pan-cytokeratin) as well as single antigen IMF samples were also prepared to quantify FRET effects and obtain reference patterns (for TOM20 and golgin), respectively.

### Experimental setup and measurements

Confocal microscopy was performed on a time-resolved microscope (MicroTime 200, PicoQuant, Germany) equipped with a fast galvo scanner (FLIMbee, PicoQuant, Germany) operated by the SymPhoTime 64 software (PicoQuant, Germany). We used two lasers in PIE mode with wavelengths of 485 nm and 561 nm (LDH-D-C-485 and LDH-D-TA-560; PicoQuant, Germany) operating at 40 MHz repetition rate and an average power between 3 µW to 6 μW measured after the objective. Samples were imaged using a 100×/1.4 NA oil-immersion objective (UPlanSApo, Olympus, USA). Sample regions of 40 μm × 40 μm (512 × 512 pixels) were imaged with an acquisition time set to 300 s with pixel dwell time equal to 5 µs.

Conventional channel mode measurements were performed using 520/35 nm and 593/20 nm bandpass filters (AHF Analysentechnik AG, Germany). For triple antigen IMF intensities in three analysis channels corresponded to following combinations of PIE mode and detection bandpass filters: Ch. 1 – excitation at 485 nm wavelength, detection spectral band of 520/35 nm, Ch. 2 – excitation at 485 nm wavelength, detection spectral band of 593/20 nm and Ch. 3 – excitation at 560 nm wavelength, detection spectral band of 593/20 nm.

The sFLIM measurements were performed with a custom-built (by PicoQuant, Germany) setup added to the MicroTime 200 system (Supplementary Fig. 2). The emitted fluorescence light was guided with a multimode fiber to the sFLIM detection system comprising of a spectrograph, an array PMT detector as well as an 8-channel TCSPC module (HydraHarp^29^ 400, PicoQuant, Germany). The fluorescence light was dispersed with a grating-based spectrograph (Shamrock SR-163 equipped with a SR1-GRT-0600-0500 grating, Andor Oxford Instruments, UK) and detected with a custom made (by PicoQuant, Germany) 16-channel PMT array detector module equipped with a gallium arsenide phosphide (GaAsP) cathode (Hamamatsu Photonics, Japan) and power supply. Corresponding to the 8 TCSPC channels, the system was configured to create 8 spectral channels with a width of 18.8 nm each covering in total a range from 490 nm to 640 nm. Information of each detected photon was stored in the time-tagged time-resolved^30^ (TTTR) data format, and further processed using a custom written sFLIM pattern-matching software^15^ written in MATLAB (MathWorks, USA). This pattern-matching based data analysis was performed on a 16-core CPU (Intel^®^ Xeon^®^ CPU E5-2680, clock speed of 2.7 GHz).

The higher sensitivity of GaAsP cathode PMT detection array together with the fully parallel 8-channel TCSPC unit allowed for high detection efficiency and faster acquisition speed. This was a major improvement over the previously reported sFLIM system^15^, where the pileup constraint due to just one TCSPC channel was a huge limitation factor precluding fast data acquisition. Furthermore, we have reduced the computation time of the sFLIM pattern-matching algorithm using a multi-core CPU from an average of 50 min per image down to 3 min (for an image of 512 × 512 pixels), equaling approximately a 16-fold increase in computation speed. Work is underway to make it even faster (less than a minute) for the method to be used and incorporated by researchers in routine experimental studies.

FCS measurements were performed using the sFLIM detection system to obtain concentrations of the dyes in aqueous solution. Dye solutions were prepared using 5 μl of each fluorescent secondary AB, “goat-anti-rabbit Alexa488”, “rabbit-anti-mouse Alexa555” and “goat-anti-mouse Alexa555”, with a 1:100 dilution in distilled water. FCS analysis was performed using the SymPhoTime 64 software. Atto488-carboxylic acid dye (ATTO-TEC GmbH, Germany) was used to obtain an effective dimension of the confocal volume, *V*_eff_ = 0.95 ± 0.10 fl with kappa, κ = 5.60 ± 0.64 using the literature value for diffusion coefficient^31^, *D *= 400 ± 1 μm^2^/s measured at 25 °C. These values were used as calibration values in FCS curve fitting analysis for all corresponding AB-dye FCS measurements. Finally, we obtained absolute concentrations of “goat-anti-rabbit Alexa488” equal to 53 ± 1 nM, “rabbit-anti-mouse Alexa555” equal to 43 ± 1 nM and “goat-anti-mouse Alexa555” equal to 50 ± 1 nM.

### Fluorescence lifetime analysis

Fluorescence decays obtained were fitted with a bi-exponential decay function, re-convolved with the measured instrument response function, using the SymPhoTime 64 software. The quality of the fits was judged from the post fitting residuals and by the reduced chi-square values. In this study, intensity weighted as well amplitude weighted average lifetimes were calculated and reported for each bi-exponential fit. Lifetime values are expressed as mean ± SD from at least three independent experiments.

### Generation of reference patterns

Single labelled TOM20 with “goat-anti-rabbit Alexa488” and golgin with “rabbit-anti-mouse Alexa555” were imaged with the described sFLIM system to generate corresponding reference patterns, whereas the FRET AB pair labelled pan-cytokeratin reference pattern was obtained from double/triple labelled samples. The sFLIM information (combining emission spectra as well as fluorescence decays corresponding to each excitation laser line) from adequately selected groups of pixels from labelled antigen were merged to generate one reference pattern. The entire procedure requires that sample preparation as well as imaging conditions are kept similar for single and multi-antigen IMF (e.g. AB incubation time, relative laser power between the two laser lines, temperature, pixel dwell time etc.).

### Bleed-through calculation

Bleed-through is defined as the contribution of photon counts after pattern-matching analysis from an unmixed channel into another unmixed channel. The bleed-through analysis was performed as proposed by Winter *et al*. (see “residual crosstalk calculation”, Materials and Methods)^32^. The calculation of pan-cytokeratin signal bleed-through into TOM20 and golgin channels was straightforward, as it was convenient to find pixel regions in the images with only a pan-cytokeratin structure. To compute the bleed-through of the TOM20 and golgin into different unmixed channels, corresponding single fluorophore-AB labelled samples were prepared for each antigen and analysed using reference patterns used during triple antigen IMF unmixing.

### Statistical analysis

GraphPad Prism 7 (Version 7.01) software was used for statistical analysis. An unpaired two-tailed Student’s *t*-test was used to determine significant changes in donor fluorophore (“goat-anti-rabbit Alexa488”) lifetime after mixing of acceptor fluorophore AB (“rabbit-anti-mouse Alexa555”). Values are expressed as mean ± SD from at least three independent experiments and the significance level ($\alpha$) of 0.05 was considered significant (Fig. 3).

## Supplementary information

Supplementary Information.

## Data Availability

The data generated for triple antigen IMF and used for analysis during this study is included in this published article and its Supplementary Information files. The sFLIM software package custom written in MATLAB can be downloaded here: https://github.com/SumeetRohilla/sFLIM.
