# Supplementary material for: Multi-target immunofluorescence by separation of antibody cross-labelling via spectral-FLIM-FRET
Source: Sci Rep. 2020 Mar 2;10:3820. doi: 10.1038/s41598-020-60877-8 (PMC7052234; doi:10.1038/s41598-020-60877-8)
Supplement: Supplementary file 1 — Supplementary Information. [file 41598_2020_60877_MOESM1_ESM.docx]

**Supplementary Information**

**Multi-target immunofluorescence by separation of antibody cross-labelling via spectral-FLIM-FRET**

Sumeet Rohilla^1, 4^, Benedikt Krämer^2^, Felix Koberling^2^, Ingo Gregor^3^, Andreas C. Hocke^4*^

^1^PicoQuant Innovations GmbH, Rudower Chaussee 29 (IGZ), 12489 Berlin, Germany.

^2^PicoQuant GmbH, Rudower Chaussee 29 (IGZ), 12489 Berlin, Germany.

^3^Third Institute of Physics, Georg-August-University, Friedrich-Hund-Platz 1, 37077 Göttingen, Germany.

^4^Charité – Universitätsmedizin Berlin, corporate member of Freie Universität Berlin, Humboldt-Universität zu Berlin, and Berlin Institute of Health, Department of Internal Medicine/Infectious Diseases and Respiratory Medicine, Charitéplatz 1, 10117 Berlin, Germany.

***Address for correspondence**

Andreas C. Hocke (MD), Charité – Universitätsmedizin Berlin, corporate member of Freie Universität Berlin, Humboldt-Universität zu Berlin, and Berlin Institute of Health, Department of Internal Medicine/Infectious Diseases and Respiratory Medicine, Charitéplatz 1, 10117 Berlin, Germany. e-mail: [andreas.hocke@charite.de](mailto:andreas.hocke@charite.de)

**Supplementary Figures**

| **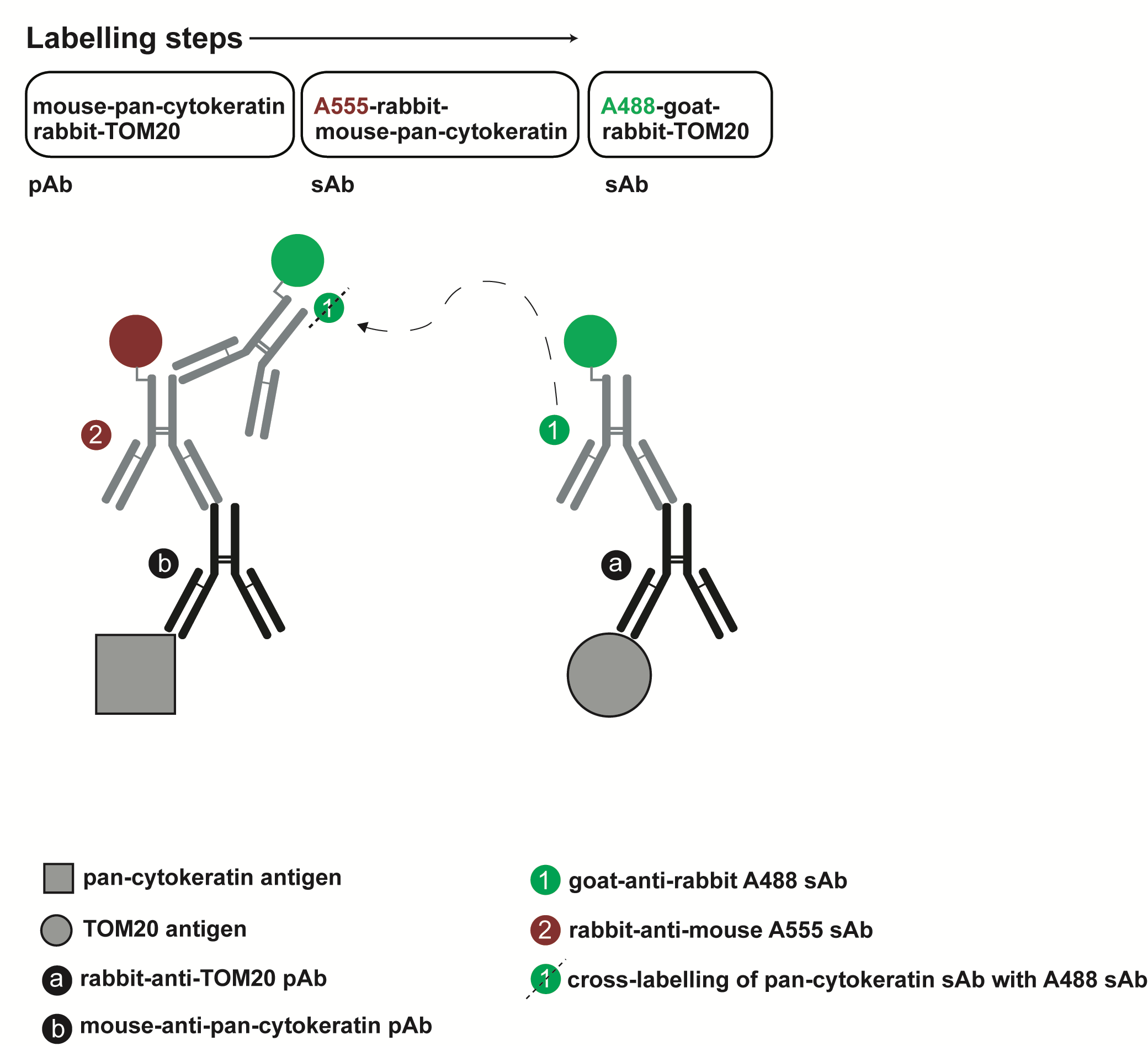** |
| --- |
| **Supplementary Figure 1. Schematic illustration of sequential labelling procedure used for cross-labelling of target antigens leading to FRET.**  A549 cells were sequentially immunolabelled for pan-cytokeratin (secondary antibody: “rabbit-anti-mouse Alexa555”) and TOM20 (secondary antibody: “goat-anti-rabbit Alexa488”) antigen) AB. This led to labelling of TOM20 with a single type of fluorophore (“goat-anti-rabbit Alexa488”) and cross-labelling of pan-cytokeratin with two different types of fluorophores (“rabbit-anti-mouse Alexa555” and “goat-anti-rabbit Alexa488”) which undergo FRET due to their spatial proximity. Shown here is 1:1 stoichiometry between secondary AB and primary AB, however, typically there are might be more than one fluorophore tagged to each secondary AB as well as more than one secondary AB attached to primary AB. |

| **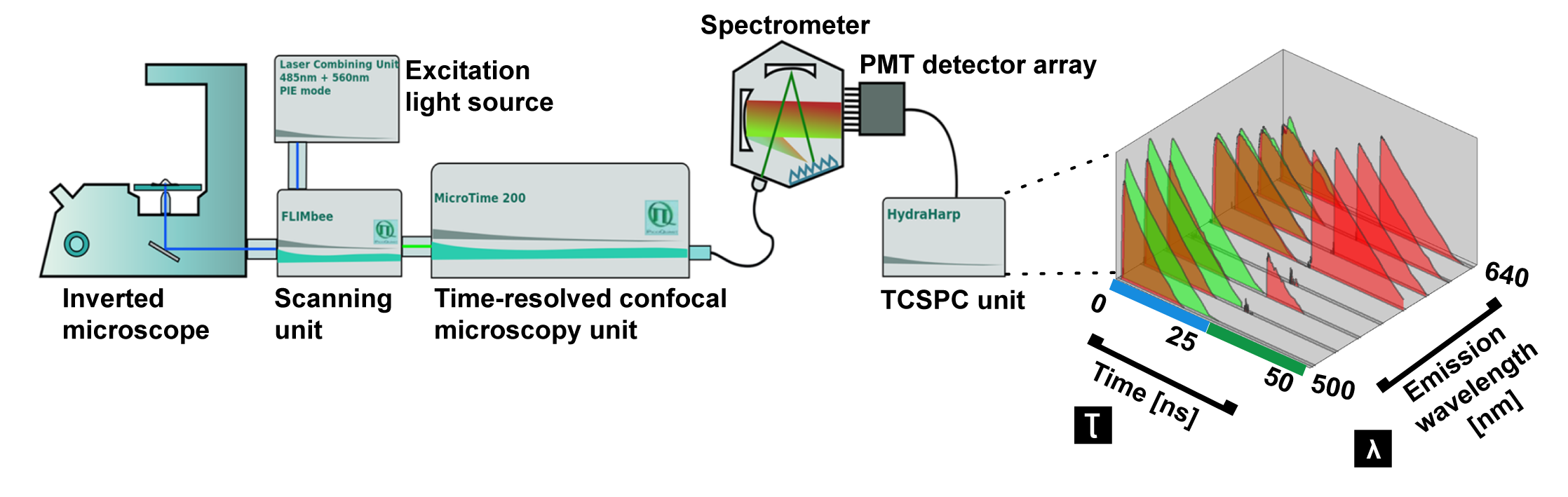** |
| --- |
| **Supplementary Figure 2.** **Schematic illustration of the spectral-FLIM experimental setup.**  Excitation is carried out by two lasers of wavelength 485 nm and 561 nm, operating in pulsed interleaved excitation mode, which were raster scanned over the sample. The emitted fluorescence light is spatially filtered with the confocal optics and then spectrally dispersed by a grating-based spectrometer. The light is detected with an 8-channel photomultiplier detector (PMT) array connected to an 8-channel TCSPC unit. For every image pixel we get a two dimensional spectral-FLIM data set consisting of a family of 8 photon arrival time histograms, one histogram for every spectral detection channel. Each arrival time histogram shows up to two maxima corresponding to photons being excited by a 485 nm laser pulse at about 2 ns and/or excited by a 561 nm laser pulse at about 27 ns. The setup allows for complete recording of the immunofluorescence signal, taking differences in the absorption as well as in the spectral and decay properties of the fluorescent labels into account (also called as reference pattern, see Materials and Methods for details). |

| **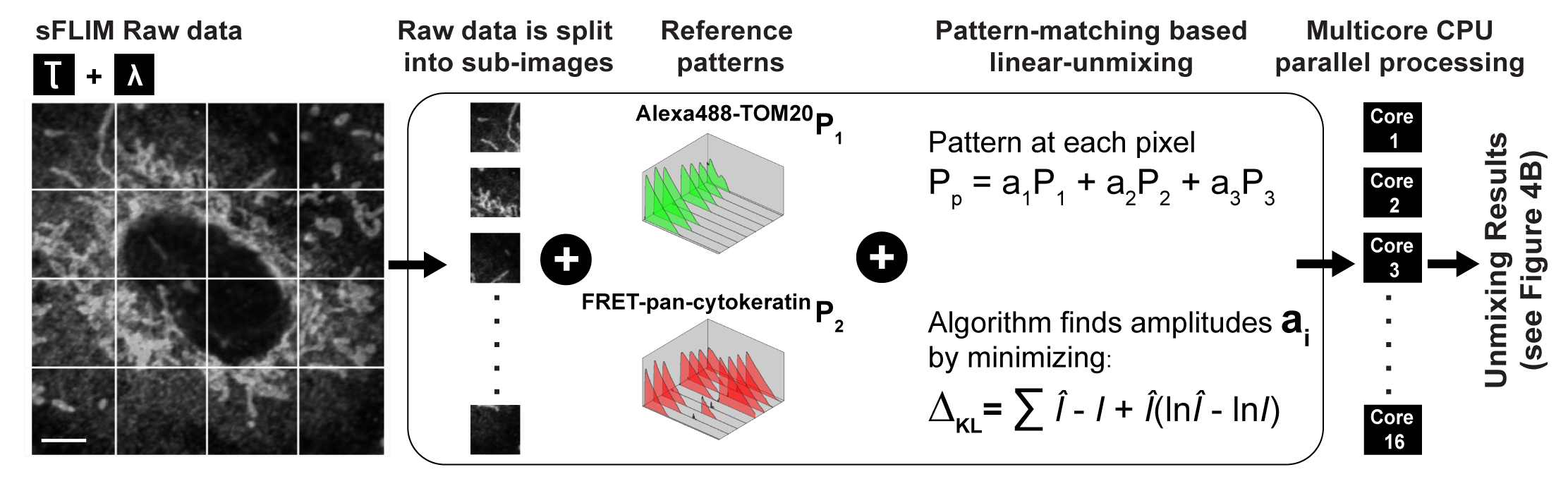** |
| --- |
| **Supplementary Figure 3. Linear-unmixing based pattern-matching approach for spectral-FLIM data analysis.**  From left to right, acquired multi-dimensional spectrally and time-resolved fluorescence lifetime imaging microscopy (spectral-FLIM or sFLIM) data is first split into sub-images. These sub-images along with a copy of reference patterns are then analysed in parallel on a multi-core CPU using a pattern-matching based linear-unmixing algorithm (see Materials and Methods for details). Here, this analysis scheme is coarsely defined as parallel batch image processing. For instance*,* a sFLIM image (combining emission spectra and fluorescence decay information per pixel) of size 512 x 512 pixels is then split into 16 sub-images and is processed on a 16-core CPU to finally provide the fluorescence contribution per pixel (in photon counts) corresponding to each immunolabelled antigen reference pattern. |

**
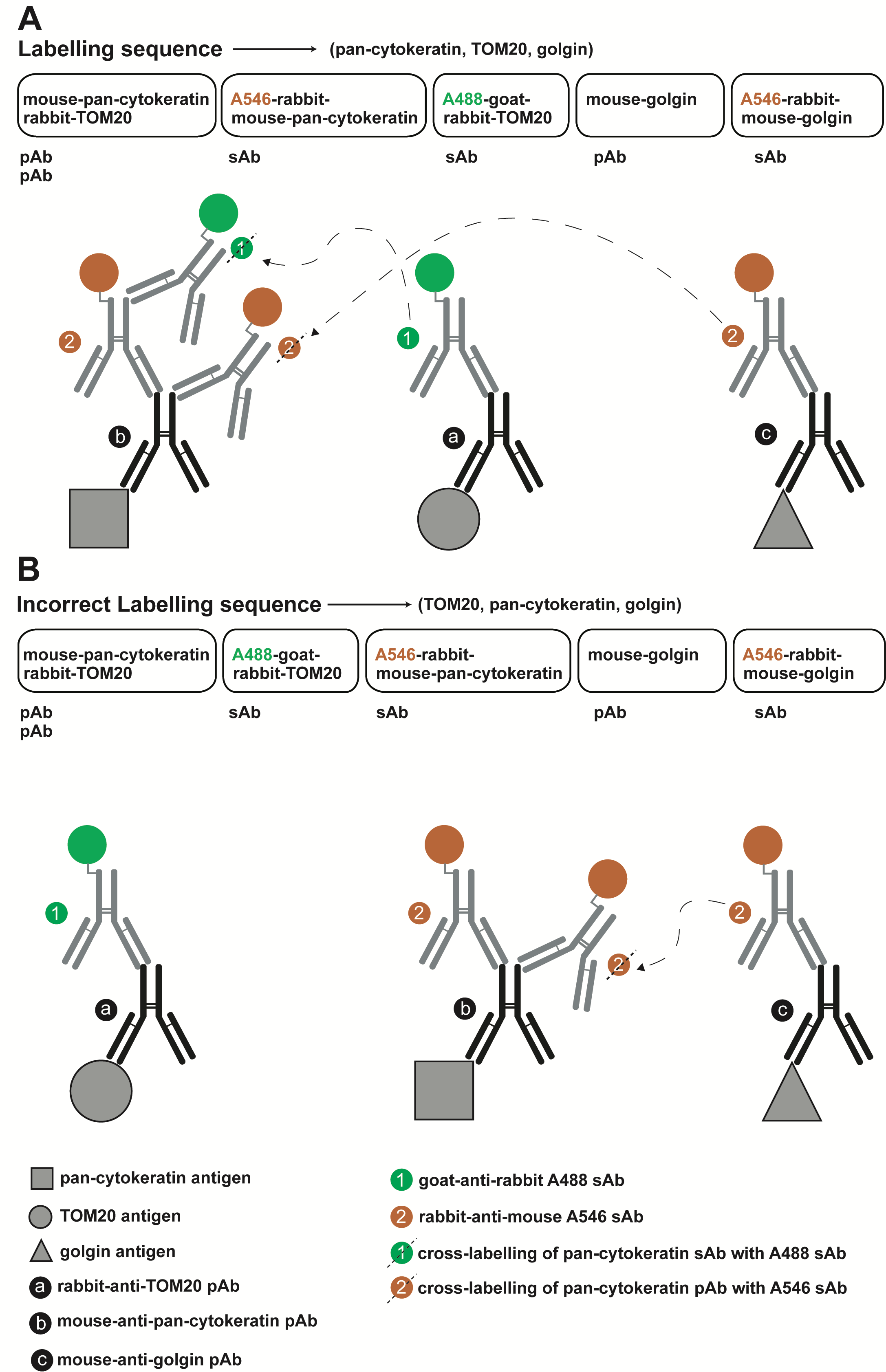
**

**Supplementary Figure 4. Labelling strategy for cross-labelling antibodies to achieve optimal FRET effects for triple antigen indirect immunofluorescence.** Shown are the two labelling strategies for triple antigen indirect immunofluorescence (IMF) on A549 cells and resulting cross-labelling antibody (AB) pairs on the target antigens. As a model, two secondary AB conjugates (“goat-anti-rabbit Alexa488” and “rabbit-anti-mouse Alexa546”), were used to achieve single labelling of TOM20 and golgin antigen as well as cross-labelling of pan-cytokeratin using two different labelling strategies. (A) Following the proposed sequential procedure, cells were immunolabelled for pan-cytokeratin (“rabbit-anti-mouse Alexa546”), TOM20 (“goat-anti-rabbit Alexa488”) and finally, golgin (“rabbit-anti-mouse Alexa546”). This led to labelling of TOM20 and golgin with a single type of fluorophore (Alexa488 and Alexa546, respectively), as well as cross-labelling of pan-cytokeratin with two different fluorophores (Alexa488 and Alexa546) undergoing FRET. By assigning a new fluorescence analysis channel to the FRET pair labelled pan-cytokeratin, quantitative separation of three antigens into independent analysis channels becomes possible by spectral-FLIM based data acquisition and pattern-matching based analysis. (B) The illustrated sequential IMF procedure results in labelling of TOM20 (“goat-anti-rabbit Alexa488”) and golgin (“rabbit-anti-mouse Alexa546”) as well as cross-labelling of pan-cytokeratin (“rabbit-anti-mouse Alexa546”). This incorrect labelling sequence leads to a labelling of both golgin and pan-cytokeratin antigen just with Alexa546 AB and renders it therefore difficult to quantitatively separate their fluorescence contribution into independent analysis channels. To make illustration easy to understand, stoichiometry of 1:1 between fluorophore and ABs is shown, however in reality, there could be on an average more than one fluorophore attached to secondary AB and/or more than one secondary AB tagged to primary AB.


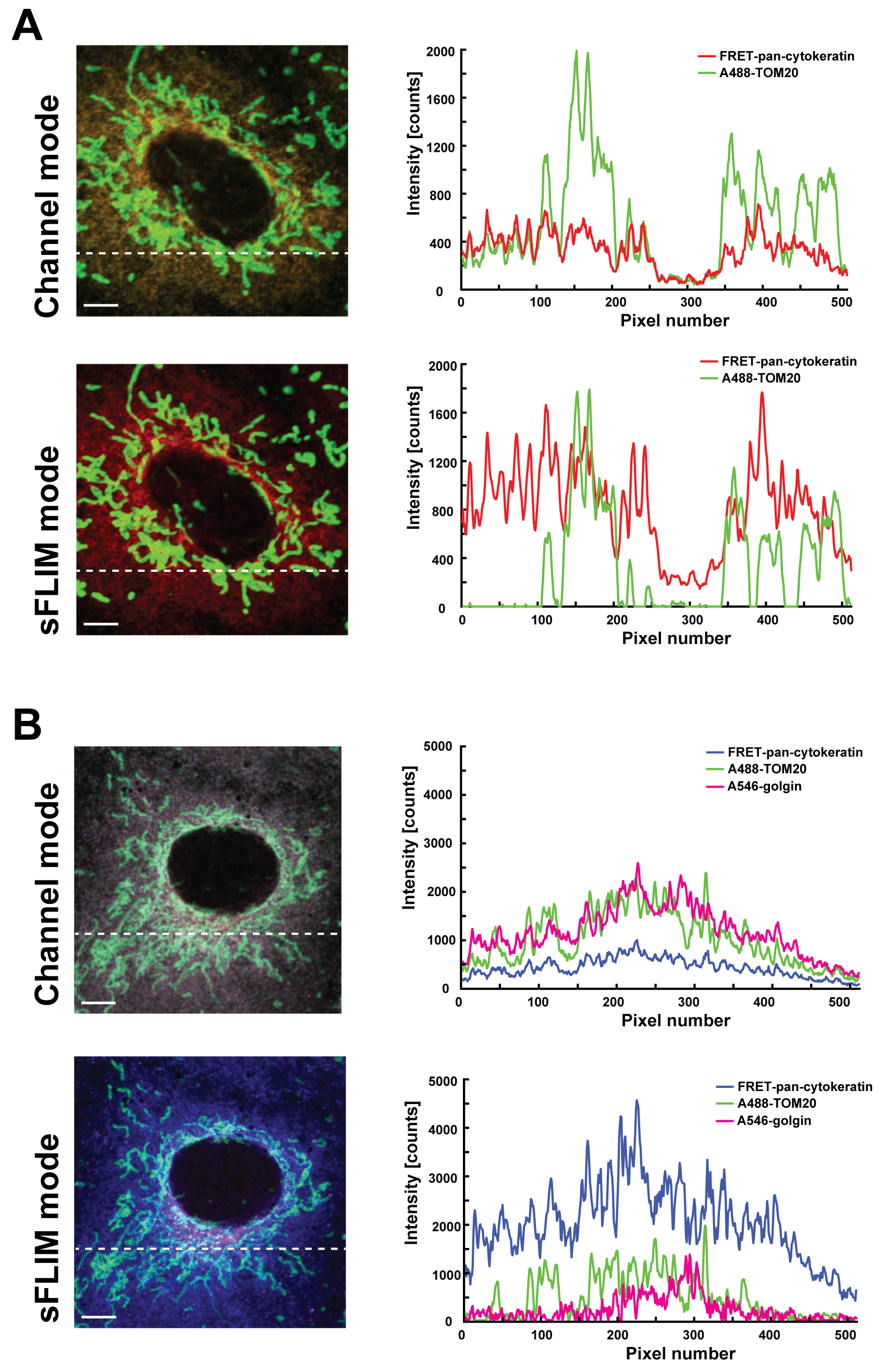


**Supplementary Figure 5. Spectral-FLIM FRET and pattern-matching based linear-unmixing enables proper channel attribution.** Shown are the line profiles of selected regions in merged images for channel mode and sFLIM based unmixing to demonstrate correct fluorescence attribution corresponding to (A) dual- and (B) triple- labelled samples using cross-labelling antibodies (ABs). The line profiles clearly show separation of signal into corresponding color channels using multi-dimensional sFLIM detection and consequent data analysis using pattern-matching algorithm, which would be impossible to achieve using conventional channel mode imaging setup; scale bars 5 μm.

**Supplementary Tables**

**A**

**B**

**C**

**Supplementary Table 1. Photophysical properties of free fluorophore-tagged antibodies and immunolabelled cellular species.**

Excitation and emission maxima as well as fluorescence lifetimes were determined using the spectral-FLIM (sFLIM) system. Obtained fluorescence decays were fitted using the SymPhoTime 64 software taking into account the measured instrument response function. Intensity weighted (𝜏_int_) as well as amplitude weighted (𝜏_amp_) average lifetimes were calculated for each bi-exponential fit. Quantification of lifetime is given as mean ± SD from three independent experiments. A549 cells were used for antigen indirect immunofluorescence. (A) Fluorescence lifetime values of free fluorophore-tagged secondary antibodies (AB) in aqueous solution. (B) Lifetime values obtained from measurements of A549 cells immunolabelled for TOM20 (“goat-anti-rabbit Alexa488”) and pan-cytokeratin with cross-labelling ABs (“goat-anti-rabbit Alexa488” and “rabbit-anti-mouse Alexa555”) exhibiting FRET. In addition, a “goat-anti-rabbit Alexa488” AB only labelled pan-cytokeratin sample was imaged to obtain the FRET donor only fluorophore lifetime value for FRET quantification on pan-cytokeratin. (C) Lifetime values obtained from measurements of A549 cells immunolabelled for TOM20 (“goat-anti-rabbit Alexa488”) AB and golgin with (“rabbit-anti-mouse Alex546”) as well as pan-cytokeratin with cross-labelling FRET AB pair (“goat-anti-rabbit Alexa488” and “rabbit-anti-mouse Alexa546”). Additionally, a “goat-anti-rabbit Alexa488” AB only labelled pan-cytokeratin sample was imaged to obtain donor fluorophore lifetime value for FRET quantification on pan-cytokeratin.
